# A New Analytic Model to Identify Lead Pollution Sources in Soil Based on Lead Fingerprint

**DOI:** 10.3390/ijerph16245059

**Published:** 2019-12-11

**Authors:** Tao Feng, Cheng-jun Wang, Yong Liu, Meng Chen, Miao-miao Fan, Zhi Li

**Affiliations:** 1 School of Management, Xi’an University of Architecture & Technology, Xi’an 710055, China; cjwang@xauat.edu.cn; 2 School of Science, Xi’an University of Architecture & Technology, Xi’an 710055, China; liuyongxa@xauat.edu.cn; 3 Tsinghua University, Haidian, Beijing 100084, China; chenm_thu@163.com; 4 Beijing University of Technology, Chaoyang, Beijing 100124, China; fanmm2@163.com; 5 California State University, San Bernardino, CA 92407, USA; liz312@coyote.csusb.edu

**Keywords:** Isotopic tracing, lead fingerprint, Gobeil’s model, pollution source, numerical analysis, Guanzhong area

## Abstract

Gobeil’s model is one of the most widely used models to identify lead (Pb) pollution sources in the environment. It is based on a set of equations involving Pb isotope fractions. Although a well-established numerical method, Gobeil’s model is often unable to provide an accurate estimation of each pollution sources’ contribution. This paper comprehensively examines the drawbacks of Gobeil’s model based on a numerical analysis and proposes a revised numerical method that provides a more accurate estimation of Pb pollution sources. Briefly, the mathematical inaccuracy of Gobeil’s model mainly lies in the misinterpretation of “lead fingerprint ratio balance.” To address this problem, the new analytic model relies on the mass balance of total lead in the contaminated sites, and uses a set of linear equations to obtain the contribution of each pollution source based on the lead fingerprint. A subsequent case study from an industrial park in Guanzhong area of Shaanxi Province in China shows that we can calculate the lead contribution rates accurately with the new model.

## 1. Introduction

The environmental pollution triggered by the enrichment effect of heavy metals has posed severe threats to human health, living environments, and agricultural production. A lot of serious health concerns have been expressed by the public and have been widely reported in the media. Lead and its compounds are common heavy metal pollutants of high toxicity. If they enter into the human body, they will destroy multiple systems including neurological, blood, digestive, kidney, cardiovascular, and endocrine [1]. Meanwhile, lead contamination in the soil can also lead to a significant reduction in the bacterial diversity, soil fertility, and soil self-purification capacity, which eventually affect the yield and quality of crops dramatically [2]. The primary sources of lead pollution are lead smelters, power plants, mining and beneficiation, burning of lead-containing fossil fuels, etc. Because lead emission sources tend to be concentrated in terms of geographical distribution, the lead pollution usually exhibits a regional pattern by these lead emission sources. In fact, field lead contamination often has multiple pollution sources, and realizing facile calculation of each pollution source’s contribution is greatly helpful for the development of practical downstream remedial strategies.

Many strategies have been established to determine the contribution of each lead pollution source to the contaminated site, including a statistical method, computer mapping method (ISOGRAM), and isotope tracing method [3,4]. Among these methods, Gobeil’s model is among the most widely used during these two decades, primarily because this model comprises a group of three linear equations that are simple to solve. This analytical model was established by Gobeil [5] in 1995 and was applied to analyze the lead pollution sources’ contribution in the sediments of St. Lawrence River estuary. Gobeil’s model relies on the fact that four stable isotopes ^204^Pb, ^206^Pb, ^207^Pb, ^208^Pb constitute the total lead in nature [6], and each lead pollution source has its own “fingerprint”—a group of combined lead isotopes with unique fractions [7]. Hence, taking advantage of these natural differences, Gobeil’s model utilizes numerical equations to resolve lead pollution sources’ contribution. A thorough scientific review could be referred to literatures by Komárek [8] and Cheng [9]. It is worth mentioning that Yu [10], Tera [11], and Tornos [12] have investigated lead isotopes’ distribution in natural media and also conducted pivotal exploratory studies, which significantly paved the way for the development of Gobeil’s model afterwards.

Since its establishment, Gobeil’s model has been applied in varying fields. For instance, Marcantonio [13], Rio-Salas [14], and Zhao [15] successfully identified the atmospheric lead pollution sources by using the Gobeil’s model. Eades [16], Bird [17,18,19], Ferrand [20], and Miller [21] studied lead pollution sources in the aquatic environment. Chiaradia [22,23], Camarero [24], Álvarez-Iglesias [25], and Luo [26] presented detailed analyses about the lead sources in soil and sediments with the Gobeil’s model. Kylander [27], Lima [28], and Anderson [29] carried out some similar research using the Gobeil’s model in the field of geology and mineral resources. In addition, Cao [30,31] and Zhao [32] have also applied the Gobeil’s model in the areas of health and food security to solve lead pollution source issues.

While the application of the Gobeil’s model is relatively widespread, the principle of its establishment has been rarely questioned. In fact, we identified a severe issue involving the misinterpretation of the lead fingerprint that might lead to huge calculation deviations of lead pollution source contribution. In this study, we thoroughly examine the Gobeil’s model and attempt to develop and validate a new analytic model for lead pollution source identification.

## 2. Materials and Methods

### 2.1. Materials

The listed materials were used to measure the lead isotope fractions with Multicollector Inductively Coupled Plasma-Mass Spectrometry (MC-ICP-MS, Neptune plus model), and they were used for digesting soil samples and purifying lead samples. The following materials in Table 1 of specific purity and manufacture brand were used.

### 2.2. Sample Digestion and Lead Measurement

The sample was digested with the following procedures:

- Around 300 mg (with the accuracy of 0.1 mg) of sample was weighed and transferred into a Teflon beaker;
- 20 mL 4% HNO_3_ was added into the sample, and it was sonicated for 40 min for digestion;
- The sample was held still for 10 min, and the clear supernatant solution then transferred into a centrifuge tube;
- 15 mL 4% HNO_3_ was added into the undissolved sample, and it was sonicated for 20 min for digestion. The sample was held still for 10 min, and the clear supernatant solution then transferred into a centrifuge tube;
- The above step was repeated;
- The collected sample was centrifuged at 4000 r/min for 15 min;
- The clear solution was loaded into the column for lead (Pb) purification.

The lead purification follows the procedures listed in Table 2.

The collected lead solution was then dried on an electrically heated plate at 160 °C, and then 200 µL of chlorazotic acid (3 drops of HCl, and 1 drop of HNO_3_) was added to the dried lead solution to dissolve any resin that came from the column. When this solution was dried, 1 more drop of HNO_3_ was added to vaporize the rest chlorazotic acid under 160 °C. The sample was then collected and loaded for further analysis.

MC-ICP-MS was used to measure the relative abundance ratio of lead isotopes in the collected samples. The following parameters were used, i.e., 1200 W power, 0.1 mL/min nebulizer gas, 0.8 L/min auxiliary gas, and 13 L/min plasma gas.

The equipment was calibrated with internal standard method. Briefly, NBS 997 Tl with ^205^Tl/^203^Tl = 2.3871 was used as an internal standard. NBS 981 (^208^Pb/^206^Pb = 2.167710, ^207^Pb/^206^Pb = 0.914750, ^206^Pb/^204^Pb = 16.9405, ^207^Pb/^204^Pb = 15.4963, ^208^Pb/^204^Pb = 36.7219) was used as standard for Pb measurement. The background Pb amount was less than 50 pg. The deviations for repeated lead isotopes measurements are ^207^Pb/^206^Pb < 0.02%, ^208^Pb/^206^Pb < 0.02%, ^206^Pb/^204^Pb < 0.04%.

## 3. Results and Discussion

### 3.1. Analysis of Gobeil’s Model

The Gobeil’s model is expressed as below in a numerical format [7]:

(1) $$\left\{ \begin{array}{l} R_{s}=f_{1}R_{1}+f_{2}R_{2}+f_{3}R_{3}\\ N_{s}=f_{1}N_{1}+f_{2}N_{2}+f_{3}N_{3}\\ f_{1}+f_{2}+f_{3}=1 \end{array}\right.$$

In Equation (1), $R_{s}$ and $N_{s}$ are defined as the abundance ratio of ^206^Pb/^207^Pb and ^208^Pb/^206^Pb, respectively. $R_{1}$, $R_{2}$, $R_{3}$ are defined as the abundance ratios of ^206^Pb/^207^Pb in the three major pollution sources, respectively. $N_{1}$, $N_{2}$, $N_{3}$ are defined as the abundance ratios of ^208^Pb/^206^Pb in the three major pollution sources, respectively. $R_{s}$, $N_{s}$, $R_{1}$, $R_{2}$, $R_{3}$, $N_{1}$, $N_{2}$, $N_{3}$ can be measured through analytical experiments, and they are regarded as known indexes in Equation (1). $f_{1}$, $f_{2}$, $f_{3}$ are defined as the weight of the three major pollution sources contributing to the contaminated site, respectively, and they are the unknown parameters to be solved. Therefore, the primary principle of the Gobeil’s model is to calculate the contribution rate of each lead pollution source by solving linear equations.

However, there is a debatable issue in the Gobeil’s model—is it appropriate to directly multiply the lead isotope abundance ratio and make a balance to that ratio of lead pollution site when modeling? In other words, in the equation of $R_{s}=f_{1}R_{1}+f_{2}R_{2}+f_{3}R_{3}$, can $f_{1}$, and $R_{1}$ be multiplied directly? What is the meaning of the result multiplied by these two parameters? In fact, based on our experiences, great deviations always occur when we use Gobeil’s model to calculate the contribution of lead pollution sources. Based on the abovementioned facts, we attempt to examine whether the equation $R_{s}=f_{1}R_{1}+f_{2}R_{2}+f_{3}R_{3}$ is always established. We at first assume that the lead isotope structures of pollution sources and contaminated samples are homogeneous and hard to change. We further demonstrated that, under the abovementioned condition, the method of taking the difference of lead isotopic abundance ratios to identify the source of lead is invalid. The relevant proof is shown in detail by Figure S1 and texts in supplementary material. The reasons of the deviation in calculating lead pollution contribution rates when using the Gobeil’s model can be explained as follows: While mass balance of lead isotopes exists between pollution sources and contaminated sites, there is no direct link between lead pollution contribution rate and the lead isotope abundance ratio. Therefore, the lead pollution contribution rates cannot be regarded as the weight of lead isotope abundance ratios of each source.

This article concerns about the mathematical inaccuracies of Gobeil’s model, and a new analytical model of lead pollution sources identification will be established based on the lead isotopes mass balance. Finally, we will conduct an empirical study from an industrial park in the Guanzhong area to verify our new model. Cheng [9] summarized the historical development of lead fingerprint and its application, and this study systematically introduced the concept of lead fingerprints, determination, lead source determination, and other typical applications of isotopes. However, it did not show any specific lead content and fingerprint concept in mathematical expressions. We attempted to describe the lead fingerprint of each lead pollution site in a mathematical way in this study. As described above, there is a natural difference among the content structures of four stable lead isotopes in lead substance. This difference is taken as the basic indicator and fingerprint that distinguishes the various sources.

We set $F=\left\{x_{1},x_{2},x_{3},x_{4}\right\}$ as the fingerprint of lead substance, where $x_{1}$ is the abundance of P204b, i.e., the percentage of isotopic P204b mass in the total mass of lead in each lead pollution source. Similarly, $x_{2}$, $x_{3}$ and $x_{4}$ are the abundance of P206b, P207b, and P208b, respectively. Moreover, Equation (2) is established.

(2) $$x_{1}+x_{2}+x_{3}+x_{4}=1$$

According to Liu [16], lead fingerprints from a specific source should be stable and unique. The lead fingerprint F defined above conforms to the relevant fingerprint stability, due to the natural differences of lead isotopes content structures in the lead substance. In practice, the determination of the lead isotope is accomplished by MC-ICP-MS, and the lead isotope ratios instead of absolute weight are usually determined. Therefore, we use the following fingerprint equation to describe the lead pollution source.

*F* = {*y*_1_, *y*_2_, *y*_3_} (3)

in which y1=P204b/P206b, y2=P206b/P207b, y3=P207b/P208b. It is worth of noting that there are more than one reduction paths. Equation (3) lists only one of the possibilities. As long as the three elements contain all of the information of set F, regardless of dimension reduction path, every F obtained are all equivalent. Therefore, in practical applications, F will be used as the lead fingerprint to resolve the lead contamination sources.

### 3.2. New Analytical Model

As the mass number of lead isotope molecules is large, and the relative differences of mass number between different lead isotope molecules are quite small, there is almost no isotopic fractionation phenomenon for lead isotope molecules. Therefore, even if the physical and chemical conditions of the environmental system significantly varied (such as metallurgy, coal, coke, etc.), the lead isotopic composition of lead substance will not change generally. According to this characteristic of lead isotopes, we develop the primary assumptions for the new model: Structural change of lead substance does not occur during the migration process, that is to say, the relative proportions of each of the stable isotopes of lead remains unchanged.

**Figure 1:**
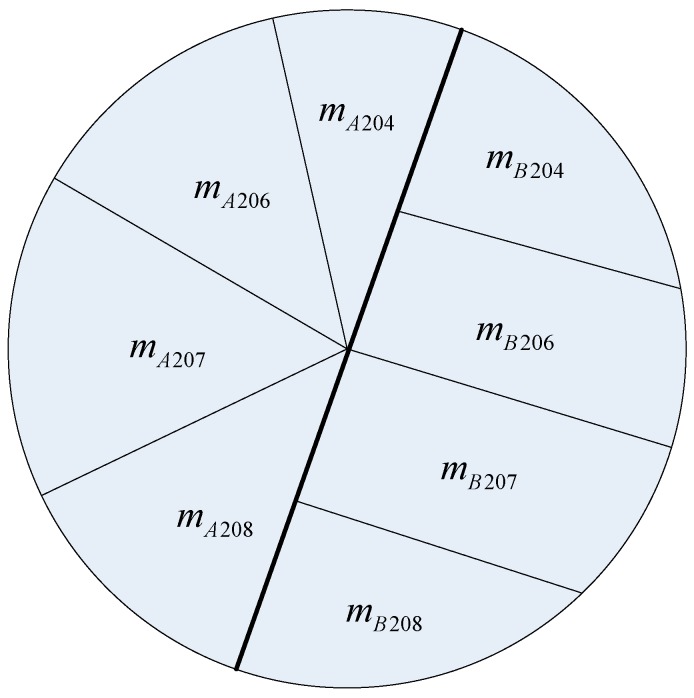
Schematic diagram of lead quality composition structure of contamination point P.

We then turned to lead pollution sources identification in the presence of multiple sources of pollution. We at first considered the simplest case in which there were only two lead pollution sources. The two lead pollution sources are labeled as A and B, respectively, and the contaminated point was denoted as P. In order to resolve the source of the lead contamination accurately, the actual target is to calculate the contribution rate of A and B for the point P, respectively. The lead composition fingerprint of contamination point P is shown in Figure 1. Let m be the total quality of lead in point P, where $m_{A}$ is the lead quality of point P which comes from source A, and $m_{B}$ is the lead quality of point P which comes from source B. As such, we get the following relationship:

(4) $$m_{A}=m_{A204}+m_{A206}+m_{A207}+m_{A208}$$

(5) $$m_{B}=m_{B204}+m_{B206}+m_{B207}+m_{B208}$$

(6) $$m=m_{A}+m_{B}$$

On this basis, the contribution rates of contamination point P ($f_{A}$, $f_{B}$) are defined as following three relationships, in detail:

(7) $$f_{A}=\frac{m_{A}}{m}=\frac{m_{A204}+m_{A206}+m_{A207}+m_{A208}}{m}$$

(8) $$f_{B}=\frac{m_{B}}{m}=\frac{m_{B204}+m_{B206}+m_{B207}+m_{B208}}{m}$$

(9) $$f_{A}+f_{B}=1$$

**Figure 2:**
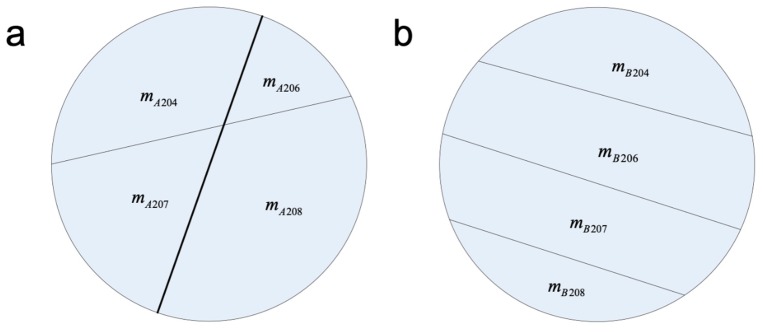
Schematic diagram of lead fingerprint in lead pollution source A (**a**) and B (**b**).

Then the lead quality composition structures of the two pollution sources A and B are discussed. They are shown in Figure 2a,b.

According to the definition of lead fingerprint previously mentioned, the lead fingerprints of sources A, B, and contaminated point P can be measured by using MC-ICP-MS. Among them, the lead fingerprint of source A is $F_{A}=\left\{k_{1},k_{2},k_{3}\right\}$, source B is $F_{B}=\left\{k_{4},k_{5},k_{6}\right\}$, and contaminated point P is $F_{P}=\left\{k_{7},k_{8},k_{9}\right\}$. The details are as follows:

(10) $$k_{1}=\frac{m_{A204}}{m_{A206}},\;k_{2}=\frac{m_{A206}}{m_{A207}},\;k_{3}=\frac{m_{A207}}{m_{A208}},\;k_{4}=\frac{m_{B204}}{m_{B206}},\;k_{5}=\frac{m_{B206}}{m_{B207}},\;k_{6}=\frac{m_{B207}}{m_{B208}},\;k_{7}=\;\frac{m_{A204}+m_{B204}}{m_{A206}+m_{B206}},\;k_{8}=\frac{m_{A206}+m_{B206}}{m_{A207}+m_{B207}},\;k_{9}=\frac{m_{A207}+m_{B207}}{m_{A208}+m_{B208}}$$

In order to obtain the contribution rates of lead source A and B, according to equations 7–10, linear equations can be established for contaminated point P as follows:

(11) $$\left\{ \begin{array}{l} f_{A}\cdot m=m_{A204}+m_{A206}+m_{A207}+m_{A208}\\ f_{B}\cdot m=m_{B204}+m_{B206}+m_{B207}+m_{B208}\\ \frac{m_{A204}}{m_{A206}}=k_{1}\\ \frac{m_{A206}}{m_{A207}}=k_{2}\\ \frac{m_{A207}}{m_{A208}}=k_{3}\\ \frac{m_{B204}}{m_{B206}}=k_{4}\\ \frac{m_{B206}}{m_{B207}}=k_{5}\\ \frac{m_{B207}}{m_{B208}}=k_{6}\\ \frac{m_{A204}+m_{B204}}{m_{A206}+m_{B206}}=k_{7}\\ \frac{m_{A206}+m_{B206}}{m_{A207}+m_{B207}}=k_{8}\\ \frac{m_{A207}+m_{B207}}{m_{A208}+m_{B208}}=k_{9}\\ f_{A}+f_{B}=1 \end{array}\right.$$

There are a total of 12 equations in Equation (11), in which $f_{A}$, $f_{B}$, $m_{A204}$, $m_{A206}$, $m_{A207}$, $m_{A208}$, $m_{B204}$, $m_{B206}$, $m_{B207}$ and $m_{B208}$ are 10 unknown parameters. $k_{i}(i=1,2\cdots 9)$ and m are known parameters which can be obtained by experiments. Because Equation (11) is a set of linear equations, the number of unknown parameters is less than the number of equations (10 < 12), the equations can be solved. The calculated two lead pollution contribution rates are as follows:

$$f_{A}=\frac{\left(k_{1}k_{2}k_{3}+k_{2}k_{3}+k_{3}+1)\times (k_{7}k_{8}k_{9}-k_{4}k_{5}k_{6}\right)}{\left(k_{1}k_{2}k_{3}+k_{2}k_{3}+k_{3}+1)\times (k_{7}k_{8}k_{9}-k_{4}k_{5}k_{6})+(k_{4}k_{5}k_{6}+k_{5}k_{6}+k_{6}+1)\times (k_{1}k_{2}k_{3}-k_{7}k_{8}k_{9}\right)}$$

$$f_{B}=\frac{\left(k_{4}k_{5}k_{6}+k_{5}k_{6}+k_{6}+1)\times (k_{1}k_{2}k_{3}-k_{7}k_{8}k_{9}\right)}{\left(k_{1}k_{2}k_{3}+k_{2}k_{3}+k_{3}+1)\times (k_{7}k_{8}k_{9}-k_{4}k_{5}k_{6})+(k_{4}k_{5}k_{6}+k_{5}k_{6}+k_{6}+1)\times (k_{1}k_{2}k_{3}-k_{7}k_{8}k_{9}\right)}$$

So far, if there are two lead pollution sources, the contribution rates can be calculated based on the newly defined fingerprint by solving Equation (11). Therefore, we have successfully realized lead pollution sources’ identification. When the number of pollution sources increases, the following conclusions can be obtained by further discussion for Equation (11): With the increase in the number of lead pollution sources, the number of linear equations in Equation (11) also increases, but the increasing speed of the number of equations is less than the increasing speed of unknown parameters. When the number of lead pollution sources is 4, the number of linear equations is exactly equal to the number of unknown parameters. This is a critical point where we can resolve the lead pollution sources. When the number of lead sources is larger than 4, the number of linear equations is less than the number of unknown parameters. Therefore, as the number of lead sources is larger than 4, the system has infinite solutions according to the basic knowledge of linear algebra. The corresponding growth of the number of equations and the number of unknown parameters is shown in Table 3. Therefore, our proposed new analytic model could successfully address up to four lead pollution sources.

### 3.3. Validation of the Proposed New Analytic Model

**Figure 3:**
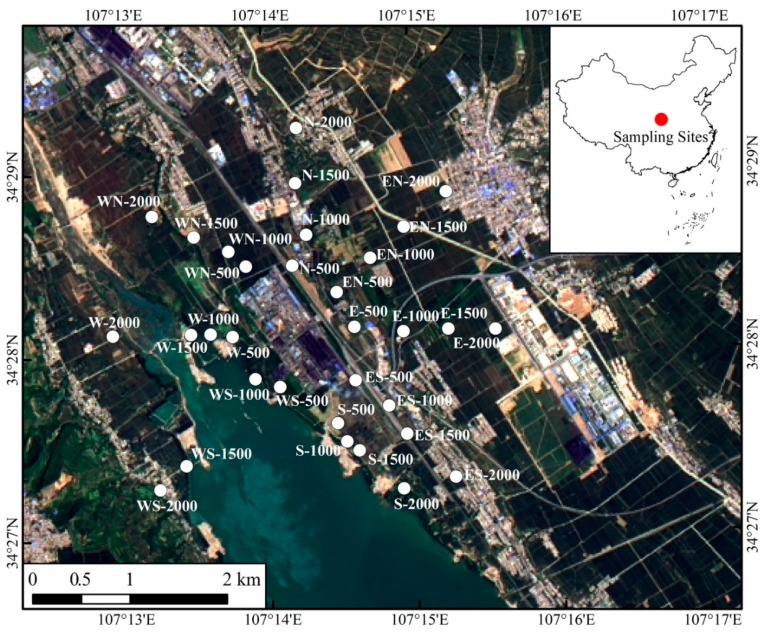
The study area and distribution of the sampling points.

To verify if our model could address the practical problems, we chose an industrial zone in the western region of the Guanzhong area, Shaanxi province, China, as the study area. The geographical environment of the study area is described below. In general, it is a narrow and long shape, and the terrain is high in the middle and low on both sides. The altitude difference is about 200 m, and the total area is about 20 km^2^. There are two reservoirs interconnected by a river in the northwest and southeast direction, respectively. The dominant wind direction is southeast wind and the secondary prevailing wind direction is north. The study area typically has a continental monsoon climate zone. The meteorological records show that the annual mean temperature is 11.2 °C, the average annual precipitation is 616.3 mm, and the average annual evaporation capacity is 1202.1 mm. The meteorological data collected in the past three years show that the mean wind speed is 2.19 m/s and the maximum wind speed is 19.0 m/s. A lead and zinc smelter and a thermal power plant are located in the region. In our study, we chose 32 sampling points in the soil. These 32 sampling points are distributed outside of the lead and zinc smelter in eight different directions (east, south, west, north, southeast, southwest, northeast, and northwest). Hence, there are four points in each direction. The distance between the point which is nearest to lead and zinc smelter and the wall is 500 m. The distribution regulation of the left three points in one direction is 1000 m, 1500 m, and 2000 m to the wall of the lead and zinc smelter, respectively. The study area and the 32 sampling points are shown in Figure 3 in detail. In addition, we have conducted several batches of sampling events for the raw ore of lead and zinc smelter, the raw coal of a coking plant and power plant, and the background value of this area. Average values of several replicates in each lead pollution sources are used. Additionally, the background value of the area is considered as a lead pollution source.

In our study, the content of heavy metal is analyzed by using Axios PW2200 (PANalytical B.V., Almelo, the Netherlands), and the Chinese first grade standard USS1-8 is used for experiment procedure controlling. The results show that the relative standard errors are all less than 10%. The lead isotope experiments are conducted in the State Key Laboratory of Loess and Quaternary Geology, Institute of Earth Environment, Chinese Academy of Sciences. Materials and methods for this assay were described in Section 2, and lead isotope ratios are measured by MC-ICP-MS. The experimental data error range is from 0.02% to 0.09%. The experimental results are shown in Table 4.

We chose raw coal of a coking plant, ore of lead and zinc smelter, raw coal of a power plant, and regional background value as four lead pollution resources. We then calculated the contribution rates of these four pollution sources based on the new analytical model. The calculation results are shown in Table 5.

It can be learned from Table 5 that the results of data points No. 14, No. 15, and No. 16 include negative values (−13.86%, −9.20%, −2.59%), which are possibly due to the interference from other lead pollution sources beyond our control. Specifically, after further study about these three points we realized that they are close to roads and villages. They are easily influenced by lead substances from automobile exhaust and burnt coal of households, the sources of which usually vary significantly. The other sites were not heavily influenced by such uncertain pollution sources. In addition, the new model is valid to the remaining 29 points. The analysis result shows the contribution rate of background value is stable and the average is 16.33%. The remaining three pollution sources have formed a complex effect on the study area, and their average contribution rates from large to small are as follows: Ore of smelting plant (35.28%), raw coal of coking plant (27.58%), and raw coal of power plant (20.81%). The contribution degree of each lead pollution source in the whole study area can be reflected based on the above sequence. Alongside this, the distribution characteristics of the contribution degree of each lead pollution source are slightly different when the direction and distance of the points are different. For example, in the south direction, the contribution rates of the lead and zinc smelter (23.75%, 28.33%, 27.81%, and 27.79%) are significantly less than the coking plant (i.e., 49.92%, 40.60%, 41.59%, and 39.00%).

## 4. Conclusions

This study reviews the previous studies on Pb pollution source identification, and then clarifies the concept of the lead fingerprints and builds a mathematical expression of lead fingerprints using the relative relationships of the four stable isotopes of lead. Based on lead fingerprints analysis, this study establishes a new pollution source analysis model and further discusses its application boundaries. Finally, a case study was conducted to verify this new model. We conclude that:

(1) Gobeil’s model is incomplete and our new established pollution source identification model with lead fingerprints can overcome the limitations of Gobeil’s model to some extent.
(2) When the number of the pollution sources is less than five, the lead contribution rates can be calculated accurately using our new model. It is not feasible to calculate lead contribution rates when the pollution sources are more than five. For example, in this study we found that the contribution rate from certain pollution sources is negative, because there is a significant interference from the other unknown pollution sources. Future research may include taking advantage of the other metal elements fingerprints to achieve more accurate calculations.
(3) Moreover, our model can be applied to identify lead pollution sources in contaminated sites where lead compound pollutant enrichment occurs, and lead substances are transported under varying meteorological, terrain, and other conditions.

## Figures and Tables

**Table 1:** Summary of materials used in this study.

Chemical	Purity	Manufacture
HNO_3_	Analytical	Millipore, Temecula, CA, USA
HF	Analytical	Honeywell Fluka, Charlotte, NC, USA
HClO_4_	Analytical	Honeywell Fluka, Charlotte, NC, USA
HBr (1 M)	Analytical	Merck, Kenilworth, NJ, USA
HCl (6 M)	Analytical	Merck, Kenilworth, NJ, USA
Milli-Q water	18.2 ΚΩ·cm	Millipore, Temecula, CA, USA
Resin for Milli-Q water	Dowex-I (200–400 mesh)	Dow, Midland, MI, USA

**Table 2:** Procedures of lead purification with run-through column.

Step	Operation	Media	Volume
1	Washing column (empty)	6.0M HCl	Full column
2	Loading resin	AG50X	Full column
3	Washing column	6.0M HCl	Full column
4	Washing column	Milli Q H_2_O	Full column
5	Washing column	6.0M HCl	Full column
6	Washing column	Milli Q H_2_O	Full column
7	Washing column	6.0M HCl	Full column
8	Washing column	Milli Q H_2_O	Full column
9	Loading sample	1.0 M HBr	Full column
10	Washing column	1.0 M HBr	Full column
11	Washing column	2.0 M HCl	Full column
12	Pb elution	6.0M HCl	Full column

**Table 3:** The possibilities of resolving the sources under condition of a plurality of lead pollution sources.

NLPS	NUPE	NELE	Equations Solvable or Not	Lead Pollution Sources Identifiable or Not
2	10	12	YES	YES
3	15	16	YES	YES
4	20	20	YES	YES
5	25	24	NO	NO
…	…	…	NO	NO

Note: NLPS: The number of lead pollution sources; NUPE: The number of unknown parameters of the equations; NELE: The number of equations of linear equations.

**Table 4:** Lead isotope measurement results of the samples.

Sample No.	Sample Code. (Azimuth-Distance)	Concentration (ppm)	^204^Pb/^206^Pb	^206^Pb/^207^Pb	^207^Pb/^208^Pb
1	E-500	54.1385	38.1028	15.6049	18.0156
2	E-1000	12.5050	37.8594	15.5957	17.8272
3	E-1500	35.7557	38.4434	15.6235	18.2839
4	E-2000	28.9605	38.6875	15.6457	18.4706
5	S-500	74.8001	37.8726	15.5961	17.8205
6	S-1000	57.0508	38.0360	15.6040	17.9401
7	S-1500	62.8752	38.0099	15.6020	17.9315
8	S-2000	53.6685	38.1233	15.6042	17.9315
9	W-500	40.6196	38.3144	15.6203	18.1589
10	W-1000	27.6219	38.5274	15.6264	18.1589
11	W-1500	33.7938	38.3724	15.6190	18.1767
12	W-2000	33.8142	38.4927	15.6209	18.2541
13	N-500	60.8520	38.1632	15.6136	18.0387
14	N-1000	27.3358	38.8615	15.6602	18.6236
15	N-1500	22.7273	38.8688	15.6584	18.6297
16	N-2000	24.4338	38.7576	15.6494	18.5355
17	ES-500	53.5663	38.2012	15.6177	18.0890
18	ES-1000	67.9129	38.0017	15.6018	17.9344
19	ES-1500	70.3960	38.0829	15.6054	17.9882
20	ES-2000	61.9352	38.0460	15.6080	17.9504
21	WS-500	44.0939	38.1154	15.6082	17.9999
22	WS-1000	22.993	38.7554	15.6505	18.5365
23	WS-1500	29.5736	38.6739	15.6443	18.4453
24	WS-2000	28.9912	38.6391	15.6394	18.4188
25	WN-500	68.5056	38.0100	15.6040	17.9505
26	WN-1000	40.8751	38.3003	15.6220	18.1678
27	WN-1500	37.7910	38.33	15.6104	18.1393
28	WN-2000	42.9699	38.2237	15.6144	18.1197
29	EN-500	26.7227	38.5385	15.6360	18.3591
30	EN-1000	29.6656	38.5777	15.6369	18.4033
31	EN-1500	29.4612	38.5998	15.6326	18.4123
32	EN-2000	24.7301	38.6627	15.6300	18.3950
33	raw coal of coking plant	184	37.2731	15.5878	17.0701
34	ore of lead and zinc smelter	27.674	38.6392	15.9509	18.4006
35	raw coal of power plant	——	38.9844	15.3821	18.3133
36	background value	——	37.8781	15.2643	18.8265

**Table 5:** Lead source analysis result basing on the new model.

Sample No.	Sample Code. (Azimuth-Distance)	$f_{\begin{array}{l} \;raw\;coal\;of\;\\ coking\;plant \end{array}}$ (%)	$f_{\begin{array}{l} ore\;\mathrm{of}\;lead\;\&\\ zinc\;smelter \end{array}}$ (%)	$f_{\begin{array}{c} \begin{array}{l} raw\;coal\;of\\ \;power\;plant \end{array} \end{array}}$ (%)	$f_{\;background\;value}$ (%)
1	E-500	36.18%	43.52%	3.69%	16.61%
2	E-1000	49.90%	23.94%	5.99%	20.16%
3	E-1500	15.61%	40.98%	24.89%	18.52%
4	E-2000	1.86%	49.57%	31.39%	17.18%
5	S-500	49.92%	23.75%	7.38%	18.95%
6	S-1000	40.60%	28.33%	13.14%	17.92%
7	S-1500	41.59%	27.81%	11.76%	18.84%
8	S-2000	39.00%	27.79%	20.69%	12.52%
9	W-500	24.24%	36.99%	21.65%	17.12%
10	W-1000	19.50%	37.25%	37.96%	5.29%
11	W-1500	22.10%	37.13%	25.56%	15.22%
12	W-2000	15.85%	39.44%	30.82%	13.89%
13	N-500	33.18%	32.62%	16.82%	17.38%
14	N-1000	−13.86%	57.25%	41.24%	15.37%
15	N-1500	−9.20%	55.88%	35.60%	17.71%
16	N-2000	−2.59%	51.92%	33.01%	17.66%
17	ES-500	57.00%	12.18%	26.97%	3.86%
18	ES-1000	41.64%	27.90%	10.96%	19.50%
19	ES-1500	37.29%	29.93%	14.17%	18.61%
20	ES-2000	39.93%	29.30%	12.84%	17.93%
21	WS-500	36.03%	30.66%	15.79%	17.53%
22	WS-1000	43.28%	31.35%	9.67%	15.70%
23	WS-1500	3.30%	48.60%	31.85%	16.25%
24	WS-2000	5.23%	47.10%	31.16%	16.51%
25	WN-500	40.72%	28.75%	10.46%	20.07%
26	WN-1000	24.16%	37.60%	19.80%	18.43%
27	WN-1500	24.70%	34.67%	25.35%	15.28%
28	WN-2000	28.05%	35.09%	17.25%	19.60%
29	EN-500	10.16%	45.05%	26.77%	18.02%
30	EN-1000	7.28%	46.43%	27.42%	18.87%
31	EN-1500	5.71%	44.72%	35.62%	13.95%
32	EN-2000	5.71%	44.72%	35.62%	13.95%
The average contribution rates (excluding 3 invalid points: 14, 15, 16)		27.58%	35.28%	20.81%	16.33%

## Supplementary Materials

The following are available online at https://www.mdpi.com/1660-4601/16/24/5059/s1, Figure S1: Schematic diagram of lead isotopic quality composition structure of sample.
