# Supplementary material for: A New Analytic Model to Identify Lead Pollution Sources in Soil Based on Lead Fingerprint
Source: Int J Environ Res Public Health. 2019 Dec 11;16(24):5059. doi: 10.3390/ijerph16245059 (PMC6950129; doi:10.3390/ijerph16245059)
Supplement: Supplementary file 1 [file ijerph-16-05059-s001.pdf]

International Journal of
Environmental Research and Public Health

MDPI

Supplementary 1

Numerical proof that the equation $R_s = f_1R_1 + f_2R_2 + f_3R_3$ in Gobeil's model is not always established

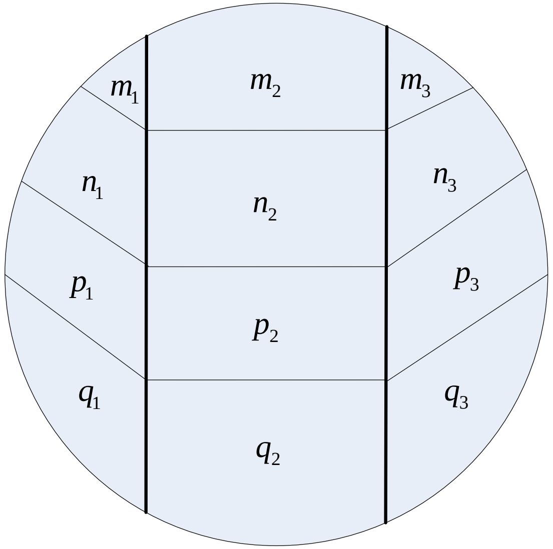

Figure S1. Schematic diagram of lead isotopic quality composition structure of sample.

In Figure S1, m_1 is the quality of ^{206}Pb which comes from pollution source 1, n_1 is the quality of ^{207}Pb which comes from pollution source 1, p_1 is the quality of ^{204}Pb which comes from pollution source 1, q_1 is the quality of ^{208}Pb which comes from pollution source 1. According to this rule, we can mark every isotopic quality which comes from pollution source 2 and pollution source 3. The variable M is labeled as the total quality of the sample. According to the definition of the isotope abundance ratio, pollution contribution rate, we can see:

$$f_1R_1 + f_2R_2 + f_3R_3 = \frac{m_1+n_1+p_1+q_1}{M}\cdot\frac{m_1}{n_1} + \frac{m_2+n_2+p_2+q_2}{M}\cdot\frac{m_2}{n_2} + \frac{m_3+n_3+p_3+q_3}{M}\cdot\frac{m_3}{n_3}, R_S = \frac{m_1+m_2+m_3}{n_1+n_2+n_3}$$

Proof:

$$f_1R_1 + f_2R_2 + f_3R_3 - R_S = \frac{m_1 + n_1 + p_1 + q_1}{M}\cdot\frac{m_1}{n_1} + \frac{m_2 + n_2 + p_2 + q_2}{M}\cdot\frac{m_2}{n_2} + \frac{m_3 + n_3 + p_3 + q_3}{M}\cdot\frac{m_3}{n_3} - \frac{m_1 + m_2 + m_3}{n_1 + n_2 + n_3}$$

$$= \frac{1}{M}\left[\frac{\mathrm{m}_1(m_1 + n_1 + p_1 + q_1)}{n_1} + \frac{\mathrm{m}_2(m_2 + n_2 + p_2 + q_2)}{n_2} + \frac{\mathrm{m}_3(m_3 + n_3 + p_3 + q_3)}{n_3}\right] - \frac{m_1 + m_2 + m_3}{n_1 + n_2 + n_3}$$

$$= \frac{1}{M}\left[\frac{\mathrm{m}_1 n_2 n_3(m_1 + n_1 + p_1 + q_1) + m_2 n_1 n_3(m_2 + n_2 + p_2 + q_2) + \mathrm{m}_3 n_1 n_2(m_3 + n_3 + p_3 + q_3)}{n_1 n_2 n_3}\right] - \frac{m_1 + m_2 + m_3}{n_1 + n_2 + n_3}$$

$$= \frac{1}{M\cdot n_1 n_2 n_3}\left[n_1 n_2 n_3(m_1 + m_2 + m_3) + m_1 n_2 n_3(m_1 + p_1 + q_1) + m_2 n_1 n_3(m_2 + p_2 + q_2) + m_3 n_1 n_2(m_3 + p_3 + q_3)\right] - \frac{m_1 + m_2 + m_3}{n_1 + n_2 + n_3}$$

$$= \frac{(n_1 + n_2 + n_3)\cdot[n_1 n_2 n_3(m_1 + m_2 + m_3) + m_1 n_2 n_3(m_1 + p_1 + q_1) + m_2 n_1 n_3(m_2 + p_2 + q_2) + m_3 n_1 n_2(m_3 + p_3 + q_3)] - M\cdot n_1 n_2 n_3\cdot(m_1 + m_2 + m_3)}{M\cdot n_1 n_2 n_3\cdot(n_1 + n_2 + n_3)}$$

Set $\alpha = (n_1 + n_2 + n_3)\cdot[n_1 n_2 n_3(m_1 + m_2 + m_3) + m_1 n_2 n_3(m_1 + p_1 + q_1) + m_2 n_1 n_3(m_2 + p_2 + q_2) + m_3 n_1 n_2(m_3 + p_3 + q_3)] - M\cdot n_1 n_2 n_3\cdot(m_1 + m_2 + m_3)$

Then $\alpha = m_1 n_1^2 n_2 n_3 + m_1 n_1 n_2^2 n_3 + m_1 n_1 n_2 n_3^2 + m_1^2 n_1 n_2 n_3 + m_1^2 n_2^2 n_3 + m_1^2 n_2 n_3^2 + m_1(p_1 + q_1) n_1 n_2 n_3 + m_1(p_1 + q_1) n_2^2 n_3 + m_1(p_1 + q_1) n_2 n_3^2 - M m_1 n_1 n_2 n_3$ +

$m_2 n_1^2 n_2 n_3 + m_2 n_1 n_2^2 n_3 + m_2 n_1 n_2 n_3^2 + m_2^2 n_1^2 n_3 + m_2^2 n_1 n_2 n_3 + m_2^2 n_1 n_3^2 + m_2(p_2 + q_2) n_1^2 n_3 + m_2(p_2 + q_2) n_1 n_2 n_3 + m_2(p_2 + q_2) n_1 n_3^2 - M m_2 n_1 n_2 n_3$ +

$m_3 n_1^2 n_2 n_3 + m_3 n_1 n_2^2 n_3 + m_3 n_1 n_2 n_3^2 + m_3^2 n_1^2 n_2 + m_3^2 n_1 n_2^2 + m_3^2 n_1 n_2 n_3 + m_3(p_3 + q_3) n_1^2 n_2 + m_3(p_3 + q_3) n_1 n_2^2 + m_3(p_3 + q_3) n_1 n_2 n_3 - M m_3 n_1 n_2 n_3$

$= \beta + \chi + \delta$

$$\beta = m_1 n_1 n_2 n_3[(n_1 + n_2 + n_3)\cdot(1 + \frac{m_1}{n_1} + \frac{p_1 + q_1}{n_1}) - M] \qquad (1)$$

$$\chi = m_2 n_1 n_2 n_3 [(n_1 + n_2 + n_3) \cdot (1 + \frac{m_2}{n_2} + \frac{p_2 + q_2}{n_2}) - M] \tag{2}$$

$$\delta = m_3 n_1 n_2 n_3 [(n_1 + n_2 + n_3) \cdot (1 + \frac{m_3}{n_3} + \frac{p_3 + q_3}{n_3}) - M] \tag{3}$$

The following is obtained by $\alpha = \beta + \chi + \delta$:

$$\alpha = n_1 n_2 n_3 \cdot [(n_1 + n_2 + n_3) \cdot m_1 \cdot (1 + \frac{m_1}{n_1} + \frac{p_1 + q_1}{n_1}) - m_1 M + (n_1 + n_2 + n_3) \cdot m_2 \cdot (1 + \frac{m_2}{n_2} + \frac{p_2 + q_2}{n_2}) - m_2 M + (n_1 + n_2 + n_3) \cdot m_3 \cdot (1 + \frac{m_3}{n_3} + \frac{p_3 + q_3}{n_3}) - m_3 M]$$

$$= n_1 n_2 n_3 \cdot \left\{ (n_1 + n_2 + n_3) \cdot \left[m_1 + \frac{m_1^2}{n_1} + \frac{m_1(p_1 + q_1)}{n_1} + m_2 + \frac{m_2^2}{n_2} + \frac{m_2(p_2 + q_2)}{n_2} + m_3 + \frac{m_3^2}{n_3} + \frac{m_3(p_3 + q_3)}{n_3} \right] - M(m_1 + m_2 + m_3) \right\}$$

$$= n_1 n_2 n_3 \cdot \left\{ (n_1 + n_2 + n_3) \cdot \left[(m_1 + m_2 + m_3) + (\frac{m_1^2}{n_1} + \frac{m_2^2}{n_2} + \frac{m_3^2}{n_3}) + (\frac{m_1(p_1 + q_1)}{n_1} + \frac{m_2(p_2 + q_2)}{n_2} + \frac{m_3(p_3 + q_3)}{n_3}) \right] - M(m_1 + m_2 + m_3) \right\}$$

$$\rightarrow f_1 R_1 + f_2 R_2 + f_3 R_3 - R_S = \frac{\alpha}{M \cdot n_1 n_2 n_3 \cdot (n_1 + n_2 + n_3)}$$

$$= \frac{\left\{ (n_1 + n_2 + n_3) \cdot \left[(m_1 + m_2 + m_3) + (\frac{m_1^2}{n_1} + \frac{m_2^2}{n_2} + \frac{m_3^2}{n_3}) + (\frac{m_1(p_1 + q_1)}{n_1} + \frac{m_2(p_2 + q_2)}{n_2} + \frac{m_3(p_3 + q_3)}{n_3}) \right] - M(m_1 + m_2 + m_3) \right\}}{M \cdot (n_1 + n_2 + n_3)} \tag{4}$$

Set $\varepsilon = n_1 + n_2 + n_3$, $\phi = m_1 + m_2 + m_3$, $\varphi = \frac{m_1^2}{n_1} + \frac{m_2^2}{n_2} + \frac{m_3^2}{n_3}$, $\gamma = \frac{m_1(p_1 + q_1)}{n_1} + \frac{m_2(p_2 + q_2)}{n_2} + \frac{m_3(p_3 + q_3)}{n_3}$

so, $$f_1R_1 + f_2R_2 + f_3R_3 - R_S = \frac{\varepsilon\cdot(\phi+\varphi+\gamma)-M\phi}{M\cdot\varepsilon} \tag{5}$$

hypothesis : $f_1R_1 + f_2R_2 + f_3R_3 - R_S = 0$,If the original formula was established ,the following conditions should be met:

$\varepsilon\cdot(\phi+\varphi+\gamma)-M\phi=0$

$(M-\varepsilon)\cdot\phi=\varepsilon\cdot(\varphi+\gamma)$

$$\frac{\phi}{\varepsilon}=\frac{\varphi+\gamma}{M-\varepsilon} \tag{6}$$

From Equation 6： $Left=\frac{\phi}{\varepsilon}=\frac{m_1+m_2+m_3}{n_1+n_2+n_3}=R_S$; $\text{right}=\frac{\varphi+\gamma}{M-\varepsilon}$;

However, $$\varphi+\gamma=\frac{m_1^{\,2}}{n_1}+\frac{m_2^{\,2}}{n_2}+\frac{m_3^{\,2}}{n_3}+\frac{m_1(p_1+q_1)}{n_1}+\frac{m_2(p_2+q_2)}{n_2}+\frac{m_3(p_3+q_3)}{n_3}$$

$$=\frac{m_1}{n_1}(m_1+p_1+q_1)+\frac{m_2}{n_2}(m_2+p_2+q_2)+\frac{m_3}{n_3}(m_3+p_3+q_3)$$

$$=R_1\cdot(m_1+p_1+q_1)+R_2\cdot(m_2+p_2+q_2)+R_3\cdot(m_3+p_3+q_3)$$

So: $$right=\frac{\phi+\gamma}{M-\varepsilon}=\frac{R_1\cdot(m_1+p_1+q_1)+R_2\cdot(m_2+p_2+q_2)+R_3\cdot(m_3+p_3+q_3)}{M-\varepsilon}$$

$$=R_1\cdot\frac{(m_1+p_1+q_1)}{M-\varepsilon}+R_2\cdot\frac{(m_2+p_2+q_2)}{M-\varepsilon}+R_3\cdot\frac{(m_3+p_3+q_3)}{M-\varepsilon} \tag{7}$$

From $$\begin{cases}\dfrac{\varphi+\gamma}{M-\varepsilon}=R_S\\[2ex] f_1R_1+f_2R_2+f_3R_3=R_S\end{cases}$$

It shows: To set up the original hypothesis, $\frac{\varphi+\gamma}{M-\varepsilon}=f_1R_1+f_2R_2+f_3R_3$ should be correct.

That is $R_1\cdot\frac{(m_1+p_1+q_1)}{M-\varepsilon}+R_2\cdot\frac{(m_2+p_2+q_2)}{M-\varepsilon}+R_3\cdot\frac{(m_3+p_3+q_3)}{M-\varepsilon}=f_1R_1+f_2R_2+f_3R_3$ (8)

Must meet the conditions: $\frac{(m_1+p_1+q_1)}{M-\varepsilon}=f_1$, $\frac{(m_2+p_2+q_2)}{M-\varepsilon}=f_2$, $\frac{(m_3+p_3+q_3)}{M-\varepsilon}=f_3$;

Because the form of the three equations above are similar , we only need to discuss $\frac{(m_1+p_1+q_1)}{M-\varepsilon}=f_1$ as follows:

$$\frac{(m_1+p_1+q_1)}{M-\varepsilon}=f_1$$

$$\frac{m_1+p_1+q_1}{M-\varepsilon}=\frac{m_1+n_1+p_1+q_1}{M}$$

Set $\eta=m_1+p_1+q_1$, transform to $\frac{\eta}{M-\varepsilon}=\frac{\eta+n_1}{M}$

That is $Mn_1=\varepsilon\eta+\varepsilon n_1$, $\frac{\varepsilon}{M}=\frac{n_1}{\eta+n_1}$

Let ε,η back, that is $\frac{n_1+n_2+n_3}{M}=\frac{n_1}{m_1+n_1+p_1+q_1}$ (9)

We can see $\frac{n_1+n_2+n_3}{M}$ is exactly ^{207}pb abundance ratio of the sample, $\frac{n_1}{m_1+n_1+p_1+q_1}$ is exactly ^{207}pb abundance ratio of the source 1, From a realistic point of view, the probability when the two abundance ratios are identical is very small.

So, $\frac{n_1+n_2+n_3}{M}=\frac{n_1}{m_1+n_1+p_1+q_1}$ is not necessarily always true, and the probability of establishment is very small.

As a result, original hypothesis $f_1R_1+f_2R_2+f_3R_3-R_S=0$ is not necessarily always true, and the probability of establishment is very small. The Gobeil's model actually is not a perfect method to resolve the pollution sources.
